# Downregulation of the tumor-suppressor miR-16 via progestin-mediated oncogenic signaling contributes to breast cancer development

**DOI:** 10.1186/bcr3187

**Published:** 2012-05-14

**Authors:** Martin A Rivas, Leandro Venturutti, Yi-Wen Huang, Roxana Schillaci, Tim Hui-Ming Huang, Patricia V Elizalde

**Affiliations:** 1 Laboratory of Molecular Mechanisms of Carcinogenesis, Instituto de Biología y Medicina Experimental (IBYME), CONICET, Vuelta de Obligado 2490, C1428ADN Buenos Aires, Argentina; 2 Department of Molecular Virology, Immunology and Medical Genetics, Comprehensive Cancer Center, The Ohio State University, 460 W 12th Ave., 43210 Columbus, OH, USA; 3 Department of Molecular Medicine/Institute of Biotechnology, Cancer Therapy and Research Center, University of Texas Health Science Center, 78229-3900 San Antonio, TX, USA; 4 Experimental Therapeutics Laboratory, Vall d'Hebron Institute of Oncology, Pg. Vall d'Hebron 119-129, 08035 Barcelona, Spain

## Abstract

**Introduction:**

Experimental and clinical evidence points to a critical role of progesterone and the nuclear progesterone receptor (PR) in controlling mammary gland tumorigenesis. However, the molecular mechanisms of progesterone action in breast cancer still remain elusive. On the other hand, micro RNAs (miRNAs) are short ribonucleic acids which have also been found to play a pivotal role in cancer pathogenesis. The role of miRNA in progestin-induced breast cancer is poorly explored. In this study we explored progestin modulation of miRNA expression in mammary tumorigenesis.

**Methods:**

We performed a genome-wide study to explore progestin-mediated regulation of miRNA expression in breast cancer. miR-16 expression was studied by RT-qPCR in cancer cell lines with silenced PR, signal transducer and activator of transcription 3 (Stat3) or c-Myc, treated or not with progestins. Breast cancer cells were transfected with the precursor of miR-16 and proliferation assays, Western blots or *in vivo *experiments were performed. Target genes of miR-16 were searched through a bioinformatical approach, and the study was focused on cyclin E. Reporter gene assays were performed to confirm that cyclin E 3'UTR is a direct target of miR-16.

**Results:**

We found that nine miRNAs were upregulated and seven were downregulated by progestin in mammary tumor cells. miR-16, whose function as a tumor suppressor in leukemia has already been shown, was identified as one of the downregulated miRNAs in murine and human breast cancer cells. Progestin induced a decrease in miR-16 levels via the classical PR and through a hierarchical interplay between Stat3 and the oncogenic transcription factor c-Myc. A search for miR-16 targets showed that the CCNE1 gene, encoding the cell cycle regulator cyclin E, contains conserved putative miR-16 target sites in its mRNA 3' UTR region. We found that, similar to the molecular mechanism underlying progestin-modulated miR-16 expression, Stat3 and c-Myc participated in the induction of cyclin E expression by progestin. Moreover, overexpression of miR-16 abrogated the ability of progestin to induce cyclin E upregulation, revealing that cyclin E is a novel target of miR-16 in breast cancer. Overexpression of miR-16 also inhibited progestin-induced breast tumor growth *in vitro *and *in vivo*, demonstrating for the first time, a role for miR-16 as a tumor suppressor in mammary tumorigenesis. We also found that the ErbB ligand heregulin (HRG) downregulated the expression of miR-16, which then participates in the proliferative activity of HRG in breast tumor cells.

**Conclusions:**

In this study, we reveal the first progestin-regulated miRNA expression profile and identify a novel role for miR-16 as a tumor suppressor in progestin- and growth factor-induced growth in breast cancer.

## Introduction

Progestins have arisen as important players in breast cancer etiology. Compelling experimental and clinical evidence points to a critical role for progesterone and the nuclear progesterone receptor (PR) in controlling mammary gland tumorigenesis [1-8]. However, the molecular mechanisms through which progesterone controls breast cancer growth are not yet fully understood. Multiple findings have shown that progestins either support sustained *in vitro *growth of breast cancer cells [2-4,8-11] or induce cells to progress through one or multiple rounds of cell division, followed by growth arrest at the G1/S phase [12]. Consistent with the proliferative role of PR, a series of G1/S cell cycle phase proteins are induced upon progestin stimulation of breast cancer cells including cyclins E and D1, c-Fos, and c-Myc [13,14]. Moreover, animal models strongly implicate PR in the genesis of breast cancer. Studies in genetically modified mice revealed that: 1) a PR knockout mouse shows dramatically reduced susceptibility to carcinogenesis [15], 2) progesterone increases genomic instability in p53 null mouse models of breast cancer [16], and 3) treatment of Brca-1-deficient mice with the anti-progestin mifepristone (RU486) prevented mammary tumorigenesis [17]. In addition, progestins exert a sustained proliferative response *in vivo *in the ER- and PR-positive C4HD model of mammary carcinogenesis induced by the synthetic progestin medroxyprogesterone acetate (MPA) in female BALB/c mice [9,11,18]. Moreover, this effect is fully abrogated by antiprogestins [19]. Notably, progesterone was recently shown to activate adult mammary stem cells within the mammary stem cell niche during the reproductive cycle, where mammary stem cells are putative targets for cell transformation events leading to breast cancer [20]. Finally, clinical observations as well as the recent extensive, randomized, and controlled Women's Health Initiative trial revealed that postmenopausal women who undergo a combined estrogen and progestin hormone replacement therapy (HRT) suffer a higher incidence of breast cancer than women who take estrogen alone [21-23]. Interestingly, the decline in breast cancer incidence seen during the last years in developed countries appears to be linked to drops in HRT use [24].

Upon progestin binding, PR translocates to the nucleus and binds to progesterone response elements (PREs) in the promoter of target genes. In addition to its direct transcriptional effects, PR activates signal transduction pathways through a rapid or nongenomic mechanism [8,25,26]. Consequently, progestins are broad genome regulators, acting either directly or indirectly on different sets of genes. Moreover, PR participates in extensive crosstalk with many cellular proteins and transcription factors. Our own findings demonstrated that progestins induce the transcriptional activation of signal transducer and activator of transcription 3 (Stat3), which is an absolute requirement for progestin-mediated breast cancer growth [18].

Micro RNAs (miRNAs) are a recently discovered class of noncoding endogenous RNAs with regulatory functions. Only in the past few years has the role of miRNAs in cancer and metastasis been identified [27-29]. Many papers have shown relationships between miRNA deregulation and different malignancies since then. In particular, it has been shown that miRNAs are aberrantly expressed in breast cancer and that the overall level of miRNA expression could clearly separate normal versus cancerous tissues [30]. Most recently, miRNAs were shown to be involved in different stages of breast cancer progression and metastasis [31-35].

miR-16 belongs to the miR-15/miR-16 cluster that is located on the noncoding gene deleted in leukemia 2 (*DLEU2*) [36]. Validated targets of miR-16 include many genes related to the control of cell-cycle progression, such as cyclin D1 [37] and cyclin E [38], among others [39-41].

At present, few works have assessed the relationship between steroid hormones and miRNAs in breast cancer [42,43]. miRNA signatures predict ER, PR and human epidermal growth factor receptor 2 (ErbB-2/neu) status in breast tumors, suggesting that differences in miRNAs are related to hormone-receptor expression [44]. It has also been shown that the overexpression of ErbB-2/neu, a member of the ErbB family of membrane receptor tyrosine kinases with a major role in breast cancer, causes an increase in the oncogenic miRNA miR-21, which confers an aggressive breast cancer phenotype via the downregulation of the metastasis suppressor protein PDCD-4 [32]. In a recent paper, estradiol was shown to regulate miRNAs, which control the estradiol response in breast cancer cells by targeting the oncogene c-Myc and the transcription factor E2F2 [45]. These authors showed that estradiol increased the expression of Dicer, the RNase responsible for releasing mature miRNA, implying that steroid hormones have a profound effect on miRNA regulation in breast cancer cells [45]. Furthermore, miR-22 has been shown to inhibit estrogen signaling by directly targeting estrogen receptor-α mRNA [46]. In addition, a causal relationship between miR-221 and miR-222 expression and resistance to the anti-estrogen drug tamoxifen has been identified in breast cancer [47,48]. As a whole, these findings suggest that steroid-modulated miRNAs are potent regulators of protein expression and cell fate that act on multiple levels in breast cancer growth, invasion, metastasis and hormone-therapy resistance.

Despite the undeniable role of progestins in breast cancer progression, progestin-mediated regulation of miRNAs has only recently been explored [49]. Bearing in mind the importance of progestins in breast cancer growth, we hypothesized that progestins may also govern a set of miRNAs that regulate the expression of genes relevant to breast cancer growth. Our findings indicate that progestins, acting through the classical PR and via Stat3 and c-Myc, downregulate miR-16, which is a potent tumor suppressor in breast cancer. Moreover, we found that miR-16 is a suppressor not only of progestin-induced breast cancer growth but also of heregulin (HRG)-induced breast cancer cell proliferation.

## Materials and methods

### Animals and tumors

Experiments were carried out using female BALB/c mice raised at the Instituto de Biología y Medicina Experimental (IBYME). Animal studies were conducted as previously described [18] in accordance with the highest standards of animal care, as outlined in the US National Institutes of Health Guide for the Care and Use of Laboratory Animals [50] and these procedures were approved by the IBYME Animal Research Committee. The C4HD tumor line displays high levels of estrogen receptor (ER) and PR, overexpresses ErbB-2 and ErbB-3, exhibits low ErbB-4 levels and lacks epidermal growth factor receptor (EGF-R) expression [2,3,18,51]. This tumor line does not express glucocorticoid or androgen receptors. Progestins exert a sustained proliferative response *in vitro *and *in vivo *in the C4HD tumor model [52].

### Reagents

MPA, RU486 and Dulbecco's modified Eagle's medium: Ham's F12 1:1 (DMEM) were purchased from Sigma-Aldrich (Saint Louis, MO, USA). Heregulin-β (HRG) was from R&D Systems (Minneapolis, MN, USA), and FCS was from Gibco Life Technologies (Carlsbad, CA, USA).

### Antibodies

The following antibodies were used for Western blots: anti-Stat3 (C-20) and anti-cyclin E (M-20) from Santa Cruz Biotechnology (Santa Cruz, CA, USA); anti-c-Myc (D84C12) from Cell Signaling (Beverly, MA, USA); anti-PR (clone hPRa7) and anti-β-actin (clone ACTN05) from Neomarkers (Freemont, CA, USA); anti-β-tubulin from Sigma-Aldrich and HRP-conjugated secondary antibodies from Vector Laboratories (Burlingame, CA, USA).

### Cell cultures, treatments and proliferation assays

Human breast cancer cell lines T47D and BT-474 were supplied by the American Type Culture Collection and maintained in DMEM + 10% FCS and in Roswell Park Memorial Institute medium (RPMI) + 10% FCS, respectively. Proliferation assays and treatments were performed in DMEM supplemented with 5% FCS that had been stripped of steroids by treatment with active charcoal (ChFCS). T47D-Y cells were a generous gift from K. Horwitz (University of Colorado Health Sciences Center, Denver, CO, USA). The tumorigenic BT-474.m1 cell line was kindly provided by D. Yu (The University of Texas MD Anderson Cancer Center, Houston, TX, USA) and was maintained in DMEM + 10% FCS.

Primary cultures of epithelial cells from C4HD tumors were performed as described [18,51]. For the proliferation assays, 1 × 10^4 ^C4HD cells/well were plated in 96-well plates and allowed to attach overnight. Cells were starved in DMEM supplemented with 0.1% ChFCS. Treatments were performed in 0.1% ChFCS with 10 nM MPA or the control vehicle (ethanol 1:1000) for 48 hours. Cell proliferation was evaluated by the incorporation of 1 μCi [^3^H]-thymidine during the last 16 hours of incubation (New England Nuclear, DuPont, Boston, MA, USA; specific activity 20 Ci/mmol) as previously described [53]. T47D cell proliferation was assessed after 24 hours of culture. Assays were performed in octuplicate. The proliferation of the C4HD cells was also assessed by counting the cells in the presence of Trypan Blue dye at 48 and 120 hours in the presence of 10 nM MPA or ethanol.

### Western blots

Lysates were prepared from cells subjected to the different treatments and proteins were resolved by SDS-PAGE as described previously [51,54,55]. Membranes were immunoblotted with the antibodies described in each experiment and filters were reprobed, after stripping, with antibodies against total β-actin or β-tubulin protein as a loading control.

### siRNA transfections

siRNAs targeting PR, Stat3 and c-Myc were synthesized by Dharmacon, Inc. (Lafayatte,

CO, USA). The following constructs were used: PR siRNA #1, 5'-AUAGGCGAGACUACAGACGUU-3'; PR siRNA #2, 5'-AAGUUCCGGAAACCUGGCAGA-3'; Stat3 siRNA #1, 5'-GGUCAAAUUUCCUGAGUUGUU-3'; Stat3 siRNA #3, 5'-CCACGUUGGUGUUUCAUAAUU-3'; c-Myc siRNA #5, 5'-GAAACGACGAGAACAGUUG-3' and c-Myc siRNA #6, 5'-CCACUCACCAGCACAACUA-3'. A nonsilencing siRNA oligonucleotide from Dharmacon that does not target any known mammalian gene was used as a negative control. Transfection of siRNA duplexes was performed using the DharmaFECT 1 transfection reagent according to the manufacturer's instructions. In every case, experiments were performed with the two different siRNA sequences for each protein [see Additional file 1], but the results obtained with only one of them are presented here.

### miRNA profiling

RNA was extracted from four independent primary cultures of epithelial cell of C4HD tumors, which were treated with 10 nM MPA or with the control vehicle (ethanol) using an miRVANA PARIS miRNA isolation kit from Ambion (Applied Biosystems, Austin, TX, USA) according to the manufacturer's instructions. For miRNA profiling, 490 ng total RNA was run on Applied Biosystems Mouse Low Density qPCR miRNA Array A and B cards. A total of 585 miRNA were surveyed. The cards were read on a 7900HT Fast Real-Time PCR System at the core lab facilities of the Comprehensive Cancer Center of Ohio State University.

### miR-16 and U6 snRNA qPCR

Levels of miR-16 were analyzed by real time quantitative RT-PCR (RT-qPCR) using a TaqMan^® ^MicroRNA assay specific for miR-16 of human or mouse origin (Assay ID 00391, miR-16 sequence: 5'-UAGCAGCACGUAAAUAUUGGCG-3') according to the manufacturer's instructions. Briefly, total RNA was extracted using miRVANA PARIS, and 100 ng of RNA was retrotranscribed to cDNA using the aforementioned assay. cDNA was amplified in an ABI7500 Real Time PCR instrument (Applied Biosystems) using specific TaqMan primers and TaqMan^® ^Universal PCR Master Mix, No AmpErase^® ^UNG (both from Applied Biosystems) to detect miR-16 and U6 snRNA (Assay ID 01973, U6 snRNA sequence: 5'-GTGCTCGCTTCGGCAGCACATATACTAAAATTGGAACGATACAGAGAAGATTAGCATGGCCCCTGCGCAAGGATGACACGCAAATTCGTGAAGCGTTCCATATTTT-3'). The latter was used as an endogenous control to correct variations in the experiment. Only mature miR-16 and U6 are detected using these assays. ΔΔCt values were used to assign a fold value, which was calculated as 2^-ΔΔCt^. All experiments were done in triplicate.

### Real time quantitative RT-PCR (RT-qPCR)

cDNA was amplified by RT-qPCR performed with an ABI Prism 7500 sequence detector using SYBR green PCR master mix (Applied Biosystems, Foster City, CA, USA). Primers with the following sequences were used to amplify a transcribed region of the cyclin E1 gene: 5'-CACCACTGAGTGCTCCAGAA-3' and 5'-CTGTTGGCTGACAGTGGAGA-3'. The full list of primers used to amplify miR-16 candidate target genes is shown in Additional file 2. qPCR was performed with 15 seconds of denaturing at 95°C followed by 40 amplification cycles of annealing and extension at 60°C for one minute.

### *In silico *analysis

A heat map of miRNA expression was built using the unsupervised clustering function in MultiExperiment Viewer version 4.4.1 [56,57]. miR-16 targets were searched using the search engine miRecords [58,59]. To narrow the list of predicted targets, a filter was applied to show miRNA target interactions predicted by at least five target-prediction programs. PREs were located on the DLEU2 promoter gene using MatInspector [60].

### Pre-miR transfection

Pre-miR precursors were obtained from Applied Biosystems and were used in accordance with the manufacturer's instructions. Briefly, 6 nM pre-miR-16 or a pre-miR-control (pre-miR-CTRL) that does not form any known mammalian miRNA, were transfected using the transfection reagent siPORT NeoFx (Ambion). After 48 hours, levels of miR-16 were augmented 2,500-fold compared with pre-miR-control-transfected C4HD cells.

### Transient transfections and luciferase reporter assay

In several experiments, T47D-Y cells were transiently transfected using the FuGENE HD transfection reagent (Roche Diagnostics Corporation, Indianapolis, IN, USA) with 1 μg of an expression plasmid previously generated and characterized by Horwitz and co-workers [61]. This plasmid encodes a PR-B engineered to contain a point mutation in a conserved cysteine in the first zinc finger of the DNA-binding domain (C587A-PR) and lacks the ability to bind to DNA. T47D-Y cells were also transfected with a mutant PR-B (PR-BmPro) engineered to contain alanines instead of three key prolines (P422A, P423A, P426A), thus abolishing PR binding to all SH3 domains and inhibiting the activation of c-Src family tyrosine kinases [26].

Luciferase constructs were bought from SwitchGear Genomics (Menlo Park, CA, USA) and contain the wild-type CCNE1 3' UTR downstream of the Promega destabilized luciferase reporter gene in the pSGG_3'UTR vector (pSGG-luc-CCNE1-3'-UTR), or an EMPTY multiple cloning site (pSGG-luc-EMPTY). Additional constructs carrying the wild-type CCNE1 3' UTR, or a minimal region from the CCNE1 3'-UTR which has a response site for miR-16 (luc-3' 1×TS), or the mutated response site for miR-16 (luc-3' mTS) were kindly provided by Dr. Vassella from the Institute for Pathology, University of Bern, Switzerland [62]. Cells were co-transfected in 12-well plates using FuGENE HD Transfection Reagent with 250 ng of pSGG-luc-CCNE1-3'-UTR or pSGG-luc-EMPTY firefly luciferase reporter vector + 10 ng of pRL-CMV, which encodes *Renilla *luciferase. After 48 hours, cells were re-transfected with either pre-miR-16 or pre-miR-CTRL following the protocol described above. Luciferase activity was measured 24 hours later (Dual Luciferase Reporter, Promega, Madison, WI, USA) using *Renilla *luciferase for normalization.

### Chromatin immunoprecipitation (ChIP) assays

ChIP was performed as described elsewhere previously [55,63], with minor modifications. Briefly, chromatin was sonicated to an average of about 500 bp. Sonicated chromatin was then immunoprecipitated by using 4 μg of the following antibodies: Anti-trimethyl-Histone H3 (Lys9) and anti-Acetyl H4 (both from Millipore, Billerica, MA, USA), anti-c-Myc (N-262, Santa Cruz Biotechnology) or an irrelevant IgG as control. The immunoprecipitate was collected by using protein A beads (Millipore, Temecula, CA, USA), which were washed repeatedly to remove nonspecific DNA binding. Chromatin was eluted from beads, and crosslinks were removed overnight at 65°C. DNA was then purified using the QIAquick PCR Purification Kit (QIAgen, Valencia, CA, USA) and quantified by real-time PCR. The following pair of primers was used: 5'-ACGGCAAAAGCTCTACAAGC-3' and 5'-GGGTCCTGCTTAGGAGAAAA-3' that amplify a genomic region encompassing the E-box located just upstream of Dleu2 exon 1A.

### *In vivo *tumor growth

C4HD cells (2 × 10^6 ^cells per mouse) were transiently transfected with the precursor of miR-16 (pre-miR-16) or with a control precursor (pre-miR-CTRL) and were then injected s.c. into animals treated with a 40 mg MPA depot in the opposite flank to the cell inoculum. Tumor volume, growth rate and growth delay were determined as described previously [18]. At specific times, tumor volumes in the different groups were compared using analysis of variance (ANOVA) followed by Tukey's test. A linear regression analysis was performed on tumor growth curves and slopes were compared using ANOVA followed by a parallelism test to assess the statistical significance of the observed differences.

BT-474.m1 cells (20 × 10^6 ^cells per mouse) were injected s.c. in NIH(S)-nude mice obtained from La Plata University (Argentina) concomitantly with a contralateral 0.72 mg estradiol pellet. After seven days tumor bearing mice (*n *= 12) were administered or not a MPA depot [64,65]. A week later, the tumors were excised and total RNA and proteins were prepared for subsequent analysis.

### Immunohistochemistry

Formalin-fixed paraffin-embedded C4HD tumors were cut with a microtome in 10 μm sections. Antigen retrieval was performed in 10 mM sodium citrate buffer pH 6 for 20 minutes at 96° to 98°C. Slides were incubated with primary antibodies anti cyclin E (M-20) (Santa Cruz Biotechnology, dilution 1:100 overnight at 4°C) or were incubated with control rabbit immunoglobulin G (IgG). Sections were subsequently incubated with the polydetector HRP system (Bio SB, Santa Barbara, CA, USA) and developed in 3-3'-diaminobenzidine tetrahydrochloride. Immunostainings were run with known positive and negative tumor controls and were blindly evaluated by a pathologist who ignored sample identity. Cyclin E expression was quantitated through the H-index, which was calculated as (% of tumor cells weakly stained) + (% of tumor cells moderately stained × 2) + (% of tumor cells strongly stained × 3) [66].

### Statistical analysis

For the proliferation assays and cell counts, differences between control and experimental groups were analyzed by ANOVA followed by Tukey's test.

## Results

### Progestins modulate miRNAs in breast cancer cells

**Figure 1:**
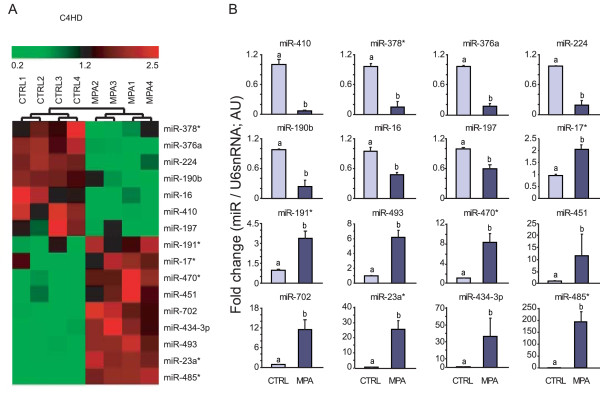
**Progestins modulate miRNAs expression in breast cancer cells**. **A**, Heat map depicting the expression profile of miRNA genes with changes ≥2 fold after 24 hours of MPA treatment. Total RNA was extracted from primary cultures of C4HD cells treated with 10 nM MPA or left untreated for 24 hours and used for miRNA profiling with the Applied Biosystems Mouse Low Density qPCR miRNA Array (*n *= 4). The small nuclear RNA U6 (U6 snRNA) was used as an endogenous control to normalize the results. **B**, Average fold changes of miRNAs significantly modulated by MPA (*P *< 0.05, *n *= 4) in primary cultures of C4HD cells. Each graph depicts the fold change of a specific miRNA in C4HD cells treated with MPA or left untreated; all values were normalized to U6 snRNA. The data shown represent the means of three independent experiments ± SEM (*P *< 0.01 for b versus a). MPA, medroxyprogesterone acetate; SEM, standard error of the mean.

To identify progestin-regulated miRNAs in breast cancer, we performed miRNA array profiling of breast cancer cells that were treated with the synthetic progestin MPA or left untreated. We used primary cultures of C4HD epithelial cells from the MPA-induced model of mammary carcinogenesis in female BALB/c mice. In these cells, progestins induce a potent and sustained mitogenic response [52]. Here, C4HD cells were treated for 24 hours with 10 nM MPA or with the control vehicle (ethanol) and total RNA was extracted. Out of the 585 mouse miRNA assayed, 350 were expressed in at least one of the two conditions [see Additional file 3]. The comparison between control- and MPA-treated cells revealed that 16 miRNAs were significantly modulated by more than two-fold (*P *< 0.05, Figure 1A), nine miRNAs were upregulated (miR-191*, miR-17*, miR- 470*, miR-451, miR-702, miR-434-3p, miR-493, miR-23a* and miR-485*) and seven were downregulated (miR-378*, miR-376a, miR-224, miR-190b, miR-16, miR-410 and miR-197) (Figure 1B). Among the differentially expressed miRNAs, we were particularly interested in miR-16, a previously reported tumor suppressor in leukemia [67,68], which was downregulated by treatment with MPA.

### Progestins downregulate miR-16 via the classical PR and a hierarchical interplay between Stat3 and c-Myc

**Figure 2:**
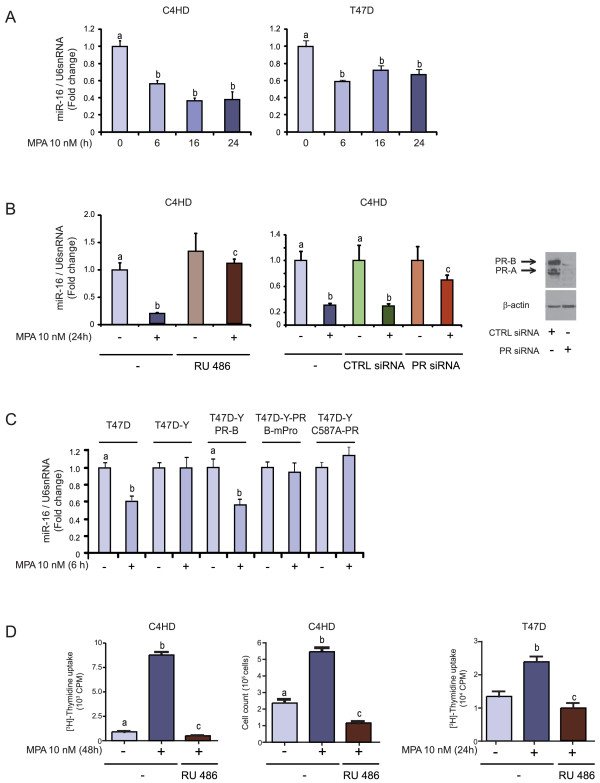
**Progestins induce miR-16 downregulation via the classical PR**. **A**, C4HD and T47D cells were treated with 10 nM MPA for the times shown. **B**, C4HD cells were either pretreated with 10 nM RU486 (left panel) or transfected with 100 nM PR and control (CTRL) siRNAs, and were then stimulated with MPA for 24 hours or left untreated (middle panel). The western blot (WB) in the right panel shows the effect of siRNAs on PR expression in C4HD cells. The experiment shown was performed with PR siRNA #1, but the same results were obtained with PR siRNA #2. **C**, T47D and T47D-Y cells were treated with MPA for the indicated time or T47D-Y cells were transiently transfected with the PR-B isoform (T47D-Y-PR-B), PR-BmPro mutant (T47D-Y-PR-BmPro cells) or the C587A-PR mutant (T47D-YC587A-PR cells) before MPA stimulation. In A to C, miR-16 expression levels were determined by RT-qPCR. The fold change of miR-16 expression levels upon MPA treatment was calculated by normalizing the absolute levels of miR-16 to those of U6 snRNA, which was used as internal control, and setting the value of untreated cells to 1. **D**, C4HD cells were treated with 10 nM MPA for 48 hours (left panel) or T47D cells were treated with 10 nM MPA for 24 hours (right panel) and the incorporation of [^3^H]-thymidine was used as a measure of DNA synthesis. The middle panel shows cell counts for C4HD cells that were treated with 10 nM MPA for 48 hours and then stained with Trypan blue dye. Experiments shown in A to D were repeated in triplicate with similar results. The data shown represent the means of three independent experiments ± SEM (*P *< 0.001 for b versus a and c versus b). MPA, medroxyprogesterone acetate; PR, progesterone receptor; SEM, standard error of the mean.

To characterize the modulation of miR-16 by MPA, we performed a time-course experiment. RNA from C4HD cells treated for 0 to 24 hours was reverse transcribed and analyzed by RT-qPCR to detect the presence of miR-16. We observed that miR-16 levels were decreased compared with control levels (Figure 2A) as early as six hours after MPA treatment and they remained low until at least 24 hours. A similar time course was observed for the human T47D breast cancer cell line (Figure 2A). Treatment of C4HD cells with the anti-progestin RU486 or silencing of PR expression using siRNAs overcame the MPA-induced miR-16 downregulation, indicating that this effect is mediated through the classical PR (Figure 2B). No modulation of miR-16 levels was observed following the sole addition of RU486 or after knockdown of PR expression in unstimulated cells (Figure 2B). These results suggest that miR-16 is regulated as part of the ligand-induced PR effects observed in breast cancer, but would not be involved in PR modulation of breast cancer growth in the absence of the ligand.

In addition, MPA was unable to modulate miR-16 in the T47D-Y cell line, a variant of the parental T47D cell line that lacks PR expression (Figure 2C). Reconstitution of PR-B levels in T47D-Y cells [55] restored MPA capacity to downregulate miR-16 (Figure 2C). In order to explore whether rapid signaling through PR and/or genomic effects participate in the MPA-downregulation of miR-16, we transfected T47D-Y cells with a PR mutant, PR-BmPro, in which three prolines (P422A, P423A, P427A) were converted to alanines (T47D-Y-PR-BmPro cells). Previous studies have defined the proline-rich domain of human PR as an absolute requirement for the interaction between progestins and c-Src [25,26] and the consequent rapid activation of signaling cascades [8,26]. Our present findings demonstrated that at least from six hours (Figure 2C) to 24 hours (data not shown) later, miR-16 levels were not regulated in response to MPA in T47D-Y-PR-BmPro cells. In addition, we restored the expression in T47D-Y cells of a PR-B mutant engineered to contain a point mutation in a conserved cysteine in the first zinc finger of the DNA binding domain (C587A), causing it to be transcriptionally crippled. Consistent with pioneering works [61], our own findings demonstrated that the C587A-PR mutant is also unable to participate in nonclassical PR tethering transcriptional mechanisms [55]. As shown in Figure 2C, MPA had no effect on miR-16 levels in T47D-Y-C587A-PR cells. These findings demonstrate the participation of both rapid (nongenomic) and transcriptional PR effects in progestin-induced miR-16 downregulation. Moreover, we found an inverse relationship between the levels of miR-16 and the proliferative state of C4HD and T47D cells. As shown in Figure 2D, MPA induces a strong proliferative response in both cell lines, which correlates with its ability to induce the downregulation of miR-16 levels (Figure 2A). These findings constitute the first piece of evidence to suggest a role for miR-16 as a tumor suppressor in progestin-induced breast cancer growth.

**Figure 3:**
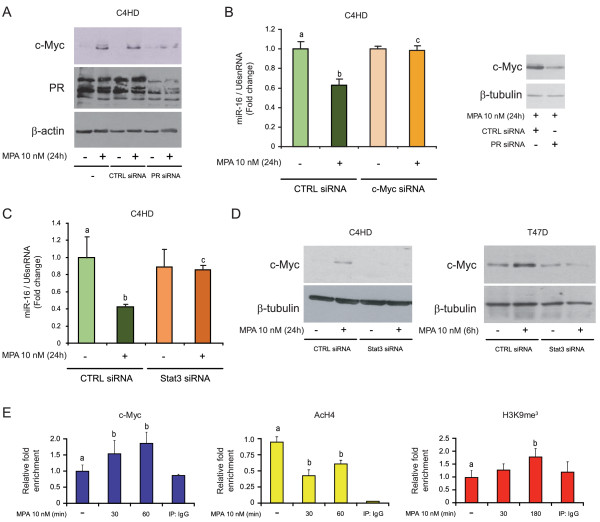
**Progestin induces miR-16 downregulation via c-Myc and Stat3**. **A**, C4HD cells were treated with MPA or transfected with PR siRNAs or CTRL siRNAs before MPA stimulation. Western blot (WB) was performed with anti-c-Myc or anti-PR antibodies and filters were reprobed with an anti-β-actin antibody. The experiment shown was performed with PR siRNA #1, but the same results were obtained with PR siRNA #2. **B**, C4HD cells were transfected with c-Myc and CTRL siRNAs and were then treated with MPA. miR-16 levels were studied by RT-qPCR, and data analysis was performed as described in Figure 2. The WB in the right side of the figure shows the effects of siRNAs on c-Myc expression in C4HD cells. The experiment shown was performed with c-Myc siRNA #5, but the same results were obtained with c-Myc siRNA #6. **C**, C4HD cells were transfected with Stat3 siRNAs or CTRL siRNAs and then treated with MPA for 24 hours. miR-16 levels were studied by RT-qPCR, and data analysis was performed as described in Figure 2. The experiment shown was performed with Stat3 siRNA #1, but the same results were obtained with PR siRNA #3. **D**, C4HD and T47D cells were transfected with Stat3 siRNAs or CTRL siRNAs before MPA stimulation and WBs were performed with anti-c-Myc antibodies. Filters were reprobed with an anti-β-tubulin antibody. The experiment shown was performed with Stat3 siRNA #1, but the same results were obtained with PR siRNA #3. **E**, Recruitment of c-Myc, and H4 acetylation (AcH4) or trimethylation of lysine 9 histone H3 (H3K9me^3^) levels at the promoter of the DLEU2 gene was studied by ChIP. Amounts of immunoprecipitated DNA were normalized to inputs and are reported relative to the untreated control group, which was set to 1 (*P *< 0.001 for b versus a). Experiments shown in A to E were repeated in triplicate with similar results. Data shown represent the means of three independent experiments ± SEM (*P *< 0.001 for b versus a and c versus b). ChIP, chromatin immunoprecipitation; MPA, medroxyprogesterone acetate; SEM, standard error of the mean.

To explore the upstream effectors involved in the MPA-mediated downregulation of miR-16, we first conducted an *in silico *analysis. We did not find canonical or half PREs at the proximal promoter of DLEU, the miR-16 host gene. This paucity indicates that miR-16 downregulation is most likely driven not by PR loading at the DLEU2 promoter, but rather by nonclassical PR tethering mechanisms. Our literature and database searches also revealed that the DLEU2 gene promoter contains two well-conserved E-box sites (CACGTG elements) which function as response elements for the oncogenic transcription factor c-Myc [36]. Therefore, we hypothesized that c-Myc, long known to be an immediate early gene for several proliferative signal cascades and whose induction by PR is well acknowledged [13,69,70], may also be an upstream effector in MPA-induced miR-16 downregulation. In accordance with previous findings in T47D cells [13,69], we found that MPA treatment also induced a significant increase in c-Myc protein levels after 24 hours of treatment in C4HD cells; this effect was abrogated by the knockdown of PR expression (Figure 3A). To explore the direct involvement of c-Myc in the molecular mechanism of MPA-induced miR-16 downregulation, we silenced c-Myc expression using siRNAs. Figure 3B (left panel) shows that knockdown of c-Myc resulted in the inhibition of MPA-induced effects on miR-16 expression.

Our previous studies of PR function demonstrated that Stat3 is a key mediator of progestin effects in breast cancer [18,71]. We found that PR induces Stat3 transcriptional activation via a nongenomic action. In addition, Stat3 activated in response to progestins is in turn directly involved in nonclassical PR transcriptional mechanisms [55,71]. Progestin-mediated modulation of miR-16 expression requires an intact PR-signaling function (Figure 2C, PR-B-mPro-transfected T47D-Y cells), the same as Stat3, and also appears to be modulated by PR-mediated transcriptional tethering mechanisms (Figure 2C, C587A-PR-transfected T47D-Y cells). Because Stat3 was found to be directly involved [55] in these mechanisms, we reasoned that Stat3 may constitute an interesting gene whose participation in progestin-mediated miR-16 expression was worth studying. Our present findings showed that indeed the knockdown of Stat3 expression resulted in a complete abrogation of MPA-induced miR-16 downregulation (Figure 3C). Stat3 function as an upstream activator of c-Myc in breast cancer has already been shown [72]. Here, we found that silencing Stat3 strongly impaired MPA-induced c-Myc upregulation (Figure 3D) in C4HD and T47D cells, for the first time demonstrating that Stat3 mediates the effects of progestin on c-Myc expression. We have previously revealed that MPA induces Stat3 expression in C4HD cells [18]. Here we found that knockdown of c-Myc expression had no effect on MPA modulation of Stat3 protein levels (data not shown). Our findings show that progestins downregulate miR-16 via the classical PR and a hierarchical interplay between Stat3 and c-Myc.

In order to further elucidate the mechanism of c-Myc induced miR-16 downregulation by MPA, we conducted chromatin immunoprecipitation assays (ChIP) on the DLEU2 promoter. Interestingly, the addition of MPA induced a two-fold increase in the recruitment of c-Myc to the E-boxes in the DLEU2 proximal promoter (Figure 3E, left panel). This result is in concordance with the ones reported by Chang *et al*. [73] in which c-Myc was shown to be recruited to E-boxes on the DLEU2 promoter. In line with a role for c-Myc as a repressor of miR-16, the addition of MPA caused a significant decrease of the levels of acetylation of histone H4 (AcH4), a chromatin modification already reported to be an activation mark for the DLEU2 locus [74] (Figure 3E, middle panel). Furthermore, we observed an increase in the levels of trimethylation of the lysine 9 in histone H3 (H3K9me3), a classical chromatin repressive mark (Figure 3E, right panel). The above results support a role for MPA as a repressor of miR-16 expression via c-Myc, inducing the recruitment of proteins with activity of chromatin remodelers which modulate gene expression.

### Pro-tumor effects of miR-16 downregulation in breast cancer are mediated by cyclin E

**Figure 4:**
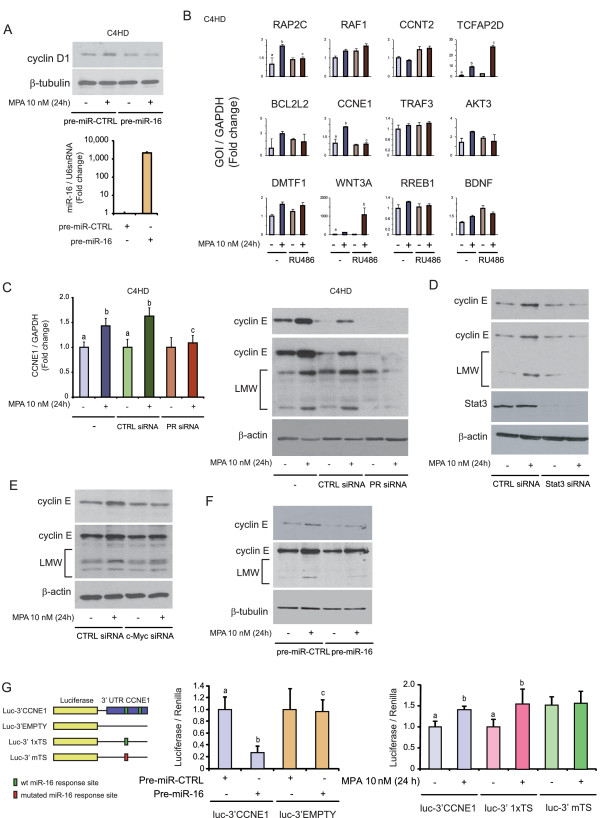
**Cyclin E is a target of miR-16 in breast cancer cells**. **A**, Upper panel, C4HD cells were transfected with pre-miR-16 or pre-miR-control (CTRL) before MPA stimulation. WB was performed with an anti-cyclin D1 antibody, and filters were reprobed with an anti-β-tubulin antibody. Bottom panel, as a control of transfection efficiency, miR-16 levels are shown in pre-miR-16-C4HD and pre-miR-CTRL-C4HD cells. **B**, C4HD cells were treated with MPA or pretreated with 10 nM RU486 before MPA stimulation, and mRNA expression levels of candidate miR-16 target genes were determined by RT-qPCR. The fold change of mRNA expression levels was calculated by normalizing the absolute levels of the gene-of-interest (GOI) mRNA to GAPDH levels, which were used as an internal control, and setting the value of untreated cells to 1. RAP2C, member of RAS oncogene family; RAF1, v-raf-1 murine leukemia viral oncogene homolog 1; CCNT2, cyclin T2; TCFAP2D, transcription factor AP-2 delta; BCL2L2, BCL2-like 2; CCNE1, cyclin E; TRAF3, TNF receptor-associated factor 3; Akt3, v-akt murine thymoma viral oncogene homolog 3; DMTF1, cyclin D binding myb-like transcription factor 1; WNT3A, wingless-type MMTV integration site family, member 3A; RREB1, ras responsive element binding protein 1; BDNF, brain-derived neurotrophic factor. **C**, C4HD cells were transfected with PR and CTRL siRNAs and then treated with MPA for 24 hours. Left panel, cyclin E mRNA levels were studied by RT-qPCR and data analysis was performed as described in Figure 2B. Right panel, WB was performed with an anti-cyclin E antibody and filters were reprobed with an anti-β-actin antibody. Longer exposures showing the expression of the low molecular weight (LMW) cyclin E isoforms are shown in the middle panel. The experiment shown was performed with PR siRNA #1, but the same results were obtained with PR siRNA #2. **D**, C4HD cells were transfected with Stat3 or CTRL siRNAs and were then treated with MPA or remained untreated. WB was performed as described in C. As a control for siRNA efficiency, the membranes were probed with an anti-Stat3 antibody. The experiment shown was performed with Stat3 siRNA #1, but the same results were obtained with PR siRNA #3. **E**, C4HD cells were transfected with c-Myc or CTRL siRNAs and then treated with MPA. WB was performed as in Figure 4C. The experiment shown was performed with c-Myc siRNA #5, but the same results were obtained with c-Myc siRNA #6. **F**, C4HD cells were transfected with pre-miR-16 or pre-miR-CTRL before MPA stimulation and WB was performed as in C. **G**, A scheme depicting the different constructions used is shown in the left panel. C4HD cells were transfected with a construct carrying the CCNE1 3' UTR cloned downstream of the firefly luciferase reporter gene (luc-3'CCNE1), middle panel, or with a construct that carried a minimal region of CCNE1 3'UTR which comprised only one of the miR-16 responding sites either wild type (luc-3' 1×TS) or mutated (luc-3' mTS), right panel. As a control, cells were transfected with a construct that lacks the 3' UTR cloned downstream of the luciferase gene (luc-3'EMPTY). Cells were co-transfected with pre-miR-16 or pre-miR-CTRL (middle panel) or treated with 10 nM MPA for 24 hours (right panel). Firefly luciferase activity was measured as described in the Methods. Renilla luciferase was used for normalization. The experiments shown in A to G were repeated in triplicate with similar results. The data shown represent the means of three independent experiments ± SEM (*P *< 0.001 for b versus a and c versus b). MPA, medroxyprogesterone acetate; PR, progesterone receptor; SEM, standard error of the mean; WB, western blot.

A variety of targets for miR-16 has already been reported, including the cell-cycle promoter cyclin D1 and the anti-apoptotic protein Bcl-2 [37]. Our own recent findings demonstrated that MPA induces the expression of cyclin D1 in C4HD cells via the assembly of a transcriptional complex between Stat3, ErbB-2 and PR, in which ErbB-2 acts as a Stat3 co-activator [55]. In the current study, we found that transfection with a precursor of miR-16 (pre-miR-16) resulted in the inhibition of MPA-induced cyclin D1 expression in C4HD cells, indicating that cyclin D1 is also a downstream target of miR-16 in breast tumor cells (Figure 4A, upper panel). As shown in Figure 4A (bottom panel), cells were efficiently transfected, reaching approximately 2,500-fold greater expression compared with C4HD cells transfected with a pre-miR-control.

To predict novel targets for miR-16, we used miRecords, a publicly available miRNA target prediction tool that integrates the predicted targets of the most commonly used search engines. To increase the stringency of the target prediction protocol, we searched for mRNAs simultaneously predicted by five or more different target-prediction programs. From a list of approximately 112 predicted interactions with mRNAs, we chose 12 with a suspected role in cancer development and progression based on the literature [See Additional file 2]. We performed RT-qPCR to amplify those mRNAs (Figure 4B) and, interestingly, we observed that CCNE1 mRNA, encoding the cell cycle regulator cyclin E, and RAP2C mRNA, encoding a member of the RAS oncogene family [75], showed a profile in response to MPA that mimicked MPA-induced proliferation; these mRNAs were also regulated inversely from miR-16. We chose to explore the regulation of CCNE1 mRNA by miR-16 due to its acknowledged role in breast cancer [76]. CCNE1 mRNA contains two highly conserved target sites for miR-16, one at position 485-491 and the other at position 241-247 of its 3' UTR. Interestingly, human and mouse CCNE1 mRNA share these target sites which suggests the importance of their preservation in gene regulation. As we already reported for C4HD cells, western blot analysis revealed the presence of the full-length, 52-kDa cyclin E isoform and a variable number of low-molecular-weight isoforms ranging in size from 35 to 50 kDa [4]. We found that MPA treatment for 24 hours induced a significant increase in CCNE1 mRNA and the expression of all protein isoforms in these cells (Figure 4C). Thus, we reasoned that cyclin E might be a true target of miR-16 in breast cancer cells. Consistent with the role of PR, Stat3 and c-Myc as upstream regulators of miR-16, the MPA-induced cyclin E increase was blocked by the silencing of PR (Figure 4C), Stat3 (Figure 4D) or c-Myc using siRNAs (Figure 4E). To validate cyclin E as a target of miR-16 action in breast cancer, we transiently transfected primary cultures of C4HD cells with pre-miR-16. As shown in Figure 4F, MPA did not induce cyclin E upregulation in miR-16-overexpressing C4HD cells, indicating that CCNE1 mRNA is indeed a direct target of miR-16. For further demonstration, we transfected C4HD cells with a construct carrying the 3' UTR of CCNE1 downstream from the luciferase gene (luc-3'CCNE1) or with a luciferase reporter gene which lacks the CCNE1 3' UTR (luc-3'EMPTY). Transfection of pre-miR-16 for 24 hours greatly decreased luciferase activity in the luc-3'CCNE1-transfected cells compared with the pre-miR-control transfected cells (Figure 4G, middle panel). Neither pre-miR-CTRL nor pre-miR-16 modified luciferase activity in the luc-3'EMPTY cells. In addition, we studied miR-16 regulation of cyclin E levels in a system in which miR-16 was not being transfected but modulated endogenously by the presence of MPA. In addition to the constructs described above, we used a construct in which only a minimal region of the CCNE1 3' UTR encompassing a miR-16 responding site was included (luc-3' 1×TS) and another in which the same site was mutated (luc-3' mTS, Figure 4G, left panel) [62]. Treatment with MPA of luc-3' CCNE1-transfected C4HD cells resulted in a significant increase of luciferase activity, in line with our hypothesis that miR-16 is a negative regulator of cyclin E (Figure 4G, right panel). In contrast, no modulation of the reporter activity was observed when C4HD cells were transfected with the luc-3' mTS and treated with MPA (Figure 4G, right panel). Noticeably, although not responsive to the endogenous changes of miR-16 levels, a higher basal luciferase activity was observed for the luc-3' mTS construct as compared to luc-3' 1 xTS or CCNE1-3'UTR, adding further evidence for a negative role of miR-16 response sites on cyclin E expression.

### miR-16 acts as a tumor suppressor in both *in vivo *and *in vitro *progestin-induced breast cancer growth

**Figure 5:**
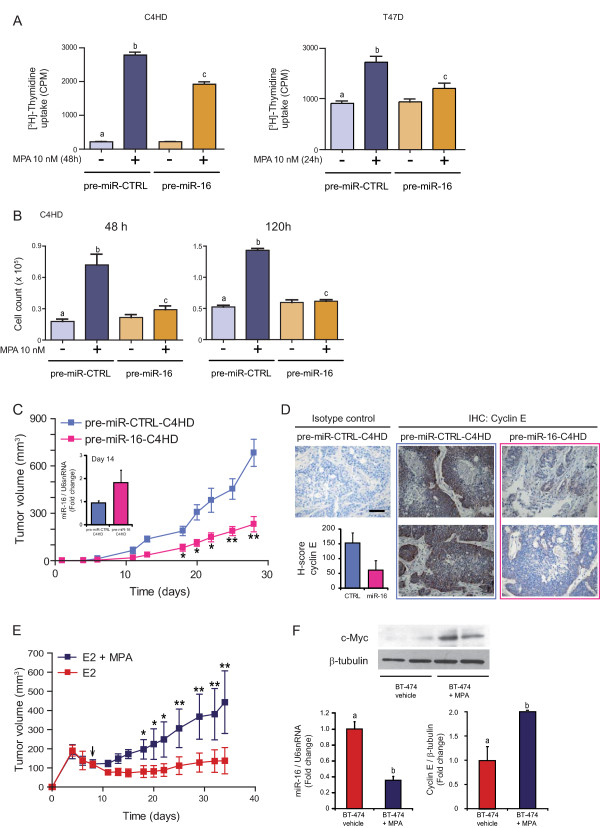
**miR-16 is a tumor suppressor in progestin-induced breast cancer growth *in vitro *and *in vivo***. **A**, miR-16 inhibits *in vitro *progestin-induced breast cancer growth. C4HD or T47D cells were transfected with pre-miR-CTRL or pre-miR-16. After 48 hours of transfection, cells were treated with 10 nM MPA or left untreated, and proliferation was measured by [^3^H]-thymidine incorporation as described in Figure 2D. **B**, C4HD cells were transfected with pre-miR-CTRL or pre-miR-16. After 48 hours, cells were treated with 10 nM MPA for 48 or 120 hours or left untreated, and proliferation was measured by cell count as described in Figure 2D. The experiments shown in A and B were repeated four times with similar results. The data shown represent the means of the data from three independent experiments ± SEM (*P *< 0.001 for b versus a and c versus b). **C**, miR-16 inhibits *in vivo *progestin-induced breast cancer growth. C4HD cells were transfected with pre-miR-CTRL or pre-miR-16 for 48 hours and then injected subcutaneously (s.c.) into BALB/c mice at 2 × 10^6 ^cells/mouse. Mice were simultaneously injected with a 40 mg MPA depot. Tumor volume was calculated as described in the Methods. Each point represents the mean volume ± SEM of six independent tumors for both experimental groups. The experiment shown in C was repeated twice with similar results. *P < 0.01 or **P < 0.001 versus control. Inset, levels of pre-miR-16 in pre-miR-CTRL-C4HD and pre-miR16-C4HD tumors were studied by RT-qPCR at day 14; data analysis was performed as described in Figure 2. **D**, Cyclin E is an *in vivo *target of miR-16. Immunohistochemistry (IHC) for cyclin E in pre-miR-CTRL-C4HD and pre-miR-16-C4HD tumors (400×). Representative images are shown. As control, IHC was performed using an irrelevant rabbit antibody. Scale bar, 50 μM. Inset, average H-score, used to quantify the levels of cyclin E in pre-miR-CTRL-C4HD and pre-miR-16-C4HD tumors. **E**, BT-474.m1 cells were injected s.c. into nude mice at 20 × 10^6 ^cells/mouse. Mice were simultaneously injected with a 0.72 mg E_2 _depot. Seven days after cell injection, half of the mice were injected with a 40 mg MPA depot (arrow). Tumor volume was calculated as described in Methods. Each point represents the mean volume ± SEM of six independent tumors for both experimental groups. *P < 0.01 or **P < 0.001 versus control. **F**, c-Myc WB was performed in whole protein extracts from BT-474 tumors growing into mice treated or not with MPA (upper panel). WB from two representative animals from each group is shown. miR-16 levels were measured in RNA from BT-474 tumors from mice treated or not with MPA (lower-left panel). Quantification of cyclin E from WB performed on whole protein extracts from BT-474 tumors from mice treated or not with MPA (lower-right panel). MPA, medroxyprogesterone acetate; SEM, standard error of the mean; WB, western blot.

To test the ability of miR-16 to counteract MPA-induced proliferation, C4HD and T47D cells were transfected with pre-miR-16 or pre-miR-CTRL, and proliferation assays were performed by measuring [^3^H]-thymidine uptake (Figure 5A) and by counting viable C4HD cells at 48 and 120 hours (Figure 5B). As shown in Figures 5A and 5B, transfection with pre-miR-16 significantly inhibited MPA-induced proliferation in C4HD and T47D cells. The results presented here indicate for the first time a role for miR-16 as a tumor suppressor in breast cancer.

We next conducted a preclinical trial to test the role of miR-16 in the MPA-induced growth of C4HD tumors *in vivo*. For this purpose, C4HD cells were transfected with pre-miR-CTRL (pre-miR-CTRL-C4HD) or pre-miR-16 (pre-miR-16-C4HD) cells and 2 × 10^6 ^cells were injected subcutaneously (s.c.) into mice treated with MPA. One representative experiment of the two performed is described here. Mice (*n *= 6) injected with pre-miR-CTRL-C4HD cells developed tumors that became palpable 12 days after inoculation. All six mice injected with pre-miR-16-C4HD cells also developed tumors, albeit with five days of tumor latency, compared with the control group. The mean volume (Figure 5C) and growth rates (Table 1) of the tumors developed from the pre-miR-16-C4HD cells were significantly lower than those of the tumors from the control group. miR-16 levels in pre-miR-16-C4HD were augmented two-fold at day 14, in comparison to pre-miR-CTRL-C4HD tumors (Figure 5C, inset). Immunohistochemistry for cyclin E revealed that pre-miR-CTRL-C4HD tumors displayed strong, mainly cytoplasmic staining for cyclin E (Figure 5D, central column and inset, H-index: 153 ± 33). In contrast, pre-miR-16-C4HD tumors stained weakly for cyclin E (Figure 5D, right column and inset, H-index: 61 ± 32), showing miR-16 efficiency *in vivo *negatively regulated cyclin E.

**Table 1:** Tumor growth rates^a^.

Treatment	Mean tumor volume (mm^3^)	Growth rate (mm^3^/day)	Growth inhibition (%)	Delay in tumor growth (days)
pre-miR-CTRL-C4HD	683.6 ± 192.2*	26.1*		
pre-miR-16-C4HD	231.1 ± 107.9^#^	9.6^#^	66.2^b^	5^b^

^a^Growth rates were calculated as the slopes of growth curves. The volume, percentage of growth inhibition, and delay in tumor growth (days) in tumors from mice injected with pre-miR16-C4HD cells relative to mice injected with pre-miR-CTRL-C4HD cells were calculated at day 28, as described in Methods. *P < 0.01, **P < 0.001 versus pre-miR-CTRL-C4HD-treated cells. ^b^Relative to pre-miR-CTRL-C4HD-treated cells, *P *< 0.001.

To extend our results to a different experimental model, we took advantage of the human BT-474.m1 breast cancer cell line which displays moderate levels of ER and PR and overexpresses the receptor tyrosine kinase ErbB-2, and which forms tumors in female nude mice. Published findings demonstrated that MPA promotes *in vivo *BT-474 tumor growth [64,65]. Therefore, 20 × 10^6 ^BT-474.m1 cells were injected s.c. in nude mice and after seven days tumor-bearing mice (*n *= 12) were administered or not a MPA depot. As expected [64], addition of MPA rescued the growth of BT-474 tumor (Figure 5E). After one week, tumors were excised and studied for miR-16, c-Myc and cyclin E levels. Interestingly, tumors growing in the presence of MPA displayed lower levels of miR-16, which correlated with higher levels of both c-Myc and cyclin E (Figure 5F). This result highlights the importance of miR-16 in progestin-promoted human breast cancer growth *in vivo*.

### The role of miR-16 as a tumor suppressor in HRG-induced breast cancer growth

**Figure 6:**
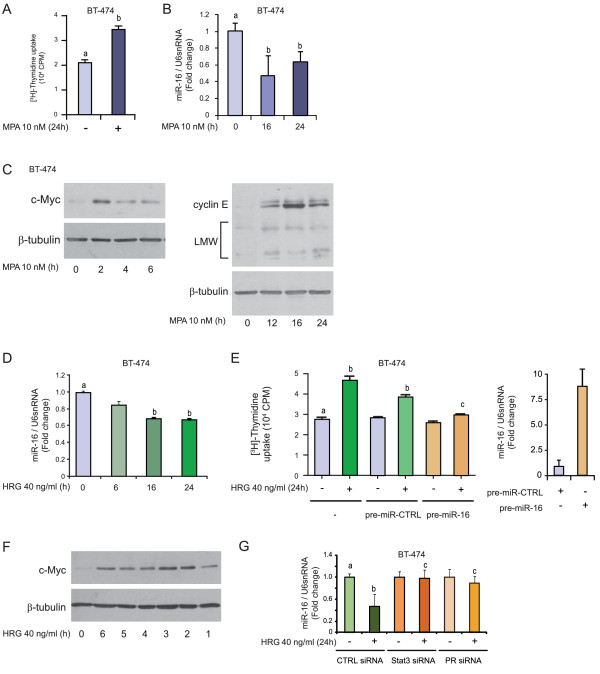
**miR-16 is a tumor suppressor in HRG-induced breast cancer growth**. **A**, BT-474 cells were treated with 10 nM MPA for 24 hours and proliferation was measured by [^3^H]-thymidine incorporation as described in Figure 2D. **B**, BT-474 cells were treated with 10 nM MPA for the times shown. miR-16 expression levels were determined by RT-qPCR, and data analysis was performed as described in Figure 2A. C, BT-474 cells were treated with MPA for the times shown and WB was performed with an anti-c-Myc antibody (left panel) or with an anti-cyclin E antibody (right panel) and filters were reprobed with an anti-β-tubulin antibody. In the WB, cyclin E LMW isoforms are shown. **D**, BT-474 cells were treated with 40 ng/ml HRG for the times shown and miR-16 levels were measured as described in Figure 2A. **E**, BT-474 cells were transfected with pre-miR-16 or pre-miR-CTRL, and proliferation was evaluated by [^3^H]-thymidine uptake as described in Figure 2D after 24 hours of HRG treatment. In the right panel, as a control for transfection efficiency, miR-16 levels are shown in pre-miR-16- and pre-miR-CTRL-transfected BT-474 cells. **F**, BT-474 cells were treated with 40 ng/ml HRG for the times shown, and WB was performed with anti-c-Myc antibody and filters were reprobed with an anti-β-tubulin antibody. **G**, BT-474 cells were transfected with Stat3, c-Myc and CTRL siRNAs and then treated with HRG for 24 hours. miR-16 levels were studied by RT-qPCR, and data analysis was performed as described in Figure 2. The experiment shown was performed with Stat3 siRNA #3 and c-Myc siRNA #5, but the same results were obtained with Stat3 siRNA #1 and c-Myc siRNA #6. Experiments shown in A to G were repeated in triplicate with similar results. The data shown represent the means of three independent experiments ± SEM (*P *< 0.001 for b versus a and c versus b). HRG, heregulin; LMW, low molecular weight; MPA, medroxyprogesterone acetate; SEM, standard error of the mean; WB, western blot.

To generalize our discovery of the role of miR-16 as a tumor suppressor, we decided to explore its involvement in the proliferation of breast cancer induced by growth factors, which along with estradiol and progestin are the major mitogens in breast cancer. In the first place, we confirmed that also in BT-474, MPA induced an increase in *in vitro *cell proliferation at 24 hours of treatment (Figure 6A) and that such increase correlated with a decrease in the expression levels of miR-16 (Figure 6B). As demonstrated for C4HD and T47D cells, the miR-16 decrease was preceded by the upregulation of c-Myc oncogene (Figure 6C, left panel) and was coincident with an increase in cyclin E expression levels (Figure 6C, right panel).

In particular, we chose to study proliferation modulated by HRG, a ligand for the ErbB family of receptor tyrosine kinases (ErbB-1, ErbB-2, ErbB-3 and ErbB-4). HRG binds ErbB-3 and ErbB-4 and recognizes EGF-R and ErbB-2 as co-receptors [77]. The roles of HRG and ErbBs in breast cancer, particularly ErbB-2, are well acknowledged [78-80]. As shown in Figure 6D, we found that HRG treatment induced a significant downregulation of miR-16 expression in BT-474 cells. This decrease in miR-16 levels, from at least 16 to 24 hours after treatment, correlated inversely with the proliferative effects of HRG at this time point (Figure 6E, left panel). Furthermore, overexpression of miR-16 resulted in significant inhibition of HRG-induced stimulation of BT-474 cell growth (Figure 6E, left panel).

Our previous findings demonstrated that HRG induces a strong Stat3 activation in breast cancer cells and, notably, that this effect is mediated via HRG co-option of PR function as a signaling molecule [53]. We here found that comparable to MPA, HRG also induced c-Myc upregulation, in a time dependent manner (Figure 6F). To explore whether HRG regulation of miR-16 was mediated via c-Myc and Stat3 expression, we silenced the expression of both proteins by the use of siRNAs and found that, comparable to our findings with MPA, silencing of Stat3 or of c-Myc both impair HRG-induced miR-16 downregulation in BT-474 cells (Figure 6G), suggesting that HRG and MPA share some of the signaling molecules in the mechanism of downregulation of miR-16.

## Discussion

Our present results indicate that the synthetic progestin MPA, a potent mitogen in C4HD and T47D cells, regulates a subset of miRNAs in mammary tumor cells including the tumor suppressor miR-16, which is downregulated. Progestins downregulate miR-16 in breast cancer cells via the classical PR and a hierarchical interplay between Stat3 and c-Myc. We showed, for the first time, that miR-16 is involved in progestin-induced tumor growth *in vitro *and *in vivo*, having the cell-cycle promoter cyclin E as a target. Remarkably, we demonstrated that miR-16 is significantly downregulated by MPA treatment in an *in vivo *setting. These results indicate a novel mechanism of progestin-induced breast cancer growth that has the potential to modulate a wide array of genes. Interestingly, we also demonstrated the involvement of miR-16 in HRG-induced breast cancer cell proliferation, confirming the ability of miR-16 to act as a tumor suppressor during breast cancer cell proliferation.

The capacity of steroid hormones to modulate miRNAs has already been described in breast cancer, mainly in estrogen-induced models [42,43]. The regulation of miRNAs by progesterone has been examined quite intensively in the uterus [81,82]. In a study by Kuokkanen *et al*., a set of 12 miRNAs were upregulated during the midsecretory phase compared with late proliferative endometrium samples [81]. In accordance with the opposite proliferation roles of progestins in the uterus and the mammary gland, none of the miRNAs that we found to be regulated by progestins in our study were also reported to be regulated in the uterus, revealing that the proliferative input of a given hormone directs miRNA modulation. Another study compared miRNA expression in leiomyomas, a benign tumor of the smooth muscle cells of the uterus, with paired normal myometrium cells. Among the downregulated miRNAs in leiomyomas, the authors identified miR-16, miR-197 and miR-224, three miRNAs we observed to be downregulated in progestin-induced breast cancer [82], highlighting the importance of miR-16 as a tumor suppressor in different cellular contexts. Notably, direct effects of progesterone on miRNA expression in normal or malignant breast cells remains poorly studied. Most recently, it was shown that MPA modulates miRNA expression in T47D cells after six hours of treatment. Progestin decreased miR-29 expression relieving the repression of the gene encoding ATPase, Na/K transporting, beta 1 polypeptide (ATP1B1), a direct PR target gene which limits migration and invasion of breast cancer cells [49].

In the past several years, miR-16 has been shown to be frequently downregulated in chronic lymphocytic leukemia [67]. It has been demonstrated that miR-16 is located in a chromosomal region commonly deleted in leukemia and that its deletion correlates with an increase in anti-apoptotic and cell-cycle-promoting proteins [83]. Nevertheless, little is known about the role of miR-16 in solid malignancies. In advanced prostate cancer, for instance, miR-15a and miR-16 are significantly downregulated, whereas the expression of BCL2, CCND1 and WNT3A is concomitantly upregulated [37]. Using experimental models, these authors showed that the restoration of miR-16 in prostate cancer cells results in growth arrest, apoptosis and in a marked regression of prostate tumor xenografts [37]. A therapeutic strategy is underway that involves the usage of atelocollagen for the delivery of synthetic miR-16 into advanced prostate tumors [84].

Recently, a few papers suggested a role for miR-16 in breast cancer, although none of them studied its modulation by steroid hormones. In accordance with the results presented here, overexpression of miR-16 was shown to suppress the self-renewal and growth of mouse mammary tumor stem cells and to sensitize MCF-7 human breast cancer cells to the chemotherapeutic drug doxorubicin [85]. Other authors demonstrated that transfection of tamoxifen-sensitive MCF-7 cells with a clinically important oncogenic isoform of ErbB-2, HER2Δ16, caused a decrease in miR-16 levels and a concomitant increase in Bcl-2 that rendered cells resistant to the treatment with tamoxifen [41]. Downregulation of miR-16 was also associated with resistance to the chemotherapeutic drug docetaxel in human breast cancer cells [86].

PR has been shown to promote breast cancer growth through rapid, nongenomic effects [8,25,26] and via its classical function as a transcription factor [87]. Our results with the mutants PR-BmPro and C587A-PR indicate that both the rapid and transcriptional effects of PR are also involved in the modulation of miRNA expression by progestins. We identified Stat3 as a key player in the downregulation of miR-16 by progestin. Our findings support the notion that Stat3 integrates the rapid and transcriptional effects of PR, leading to a decrease in miR-16 levels. Thus, rapid PR signaling is conceivably required to activate Stat3, which would then modulate the transcriptional function of PR to repress miR-16 expression. In support of this hypothesis, we have previously shown that the rapid effects of PR mediate Stat3 transcriptional activation in breast tumors [18] and that activated Stat3 in turn participates in the transcriptional mechanisms of PR that drive mammary tumor growth [71].

The c-Myc oncogenic transcription factor is pathologically activated in many human malignancies [88]. c-Myc is known to directly upregulate a pro-tumorigenic group of miRNAs, known as the miR-17-92 cluster, which acts at multiple levels of tumor progression [89]. In a pioneering study by Mendell *et al*., c-Myc regulated a much broader set of miRNAs than previously anticipated. Unexpectedly, the predominant consequence of c-Myc activation was shown to be the widespread repression of miRNA expression, probably as a direct result of c-Myc binding to miRNA promoters [73,89]. Interestingly, miR-16 was among the miRNAs repressed by c-Myc. Those results demonstrated that extensive reprogramming of the miRNA transcriptome by c-Myc contributes to tumorigenesis. Ours is the first study to demonstrate an absolute requirement for Stat3 during the well-known process of c-Myc upregulation induced by progestins [13,69]. Moreover, a recent study demonstrates that c-Myc induces the recruitment of the histone deacetylase 3 to the DLEU2 locus, causing the decrease of AcH4 and, hence, repression of miR-16 [74]. Our results are in line with the mentioned study and, in addition to the decrease of AcH4, we also showed the increase of H3K9me^3^, a general chromatin repression mark.

**Figure 7:**
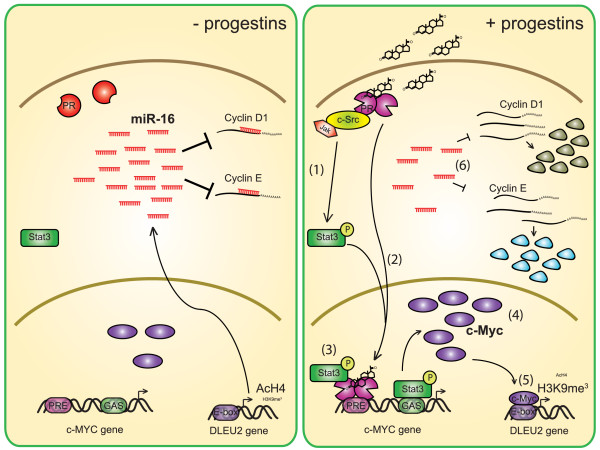
**Model of MPA-induced miR-16 downregulation and cell-cycle control**. In the absence of progestin stimulation, steady state levels of miR-16 repress the translation of key mRNAs required for cell-cycle progression, such as cyclin D1 and cyclin E mRNAs (left panel). Binding of progestins to PR (right panel) induces Stat3 activation via the activation of c-Src and Jak kinases (Step 1) [18]. At the same time, progestin-activated PR translocates to the nucleus where it binds to PREs, such as the c-Myc PRE (Step 2) [69]. In addition, Stat3 migrates to the nucleus and binds to its response elements (GAS sites) and is also known to act as a PR co-activator (Step 3) [71]. The latter event results in the upregulation of the oncogenic transcription factor c-Myc (Step 4), which represses miR-16 expression by binding to E-box response elements and inducing chromatin remodeling (decrease of AcH4 and increase of H3K9me^3^, Step 5) [36,73,74]. Decreased levels of miR-16 would result in an increased expression of its targets, including cyclin D1 and cyclin E, and would lead to cell growth (Step 6). PR, progesterone receptor; PREs, progesterone response elements.

Figure 7 illustrates our proposed model of progestin-mediated regulation of miR-16 expression leading to breast cancer growth, based on our previous and present findings. In this model, the rapid action of progestin induces the phosphorylation of Stat3 via c-Src and Jaks, as we showed previously [18] (Step 1). Upon progestin binding, PR migrates to the nuclear compartment and binds to a PRE at the c-Myc promoter, as widely acknowledged [69] (Step 2). We propose that the requirement for both the rapid and genomic functions of PR during the regulation of miR-16 expression by progestins, as demonstrated in our study, may be explained by the fact that after being rapidly activated by PR, Stat3 is recruited, along with PR, to the PRE at the c-Myc promoter, where it acts as a PR co-activator (Step 3). Our most recent findings support this hypothesis. We in fact demonstrated that Stat3 acts as a PR co-activator at the promoters of the mouse mammary tumor virus (MMTV) and the endogenous gene bcl-X in breast cancer cells [71], raising the possibility that the role of Stat3 as a PR co-activator is a general mechanism for the modulation of the transcriptional effects of PR. However, the c-Myc promoter also contains Stat3 response elements (GAS sites); therefore, Stat3 may also possibly induce c-Myc expression through its role as a transcription factor (Step 4). The PR/Stat3 transcriptional complex and possibly also Stat3 bound to GAS sites, induce the expression of c-Myc, which would in turn associate with the DLEU2 promoter and repress the expression of miR-16 (Step 5). The role of c-Myc as a transcriptional repressor of miR-16 has previously been shown [36,73] and we here also demonstrate its involvement in mR-16 downregulation upon progestin treatment. In addition, c-Myc recruitment to the DLEU2 triggers a chromatin remodeling program which results in a decrease of AcH4 and an increase in H3K9me, which ultimately translate into repression of the DLEU2 locus and miR-16 decrease. This decrease in the levels of intracellular miR-16 would result in increased expression of its targets, including cyclin D1 and E, and would lead to cell growth (Step 6).

Cyclin E is a critical protein for the G0 to G1 entry in the cell cycle and its role in breast cancer is well acknowledged [76,90-92]. Moreover, cyclin E overexpression has been recently demonstrated to confer trastuzumab resistance in ErbB-2-overexpressing breast cancer [93]. In addition to the full length isoform of cyclin E, a variety of low molecular weight (LMW) isoforms are present in breast cancer. The presence of these LMW isoforms was demonstrated to strongly correlate with decreased survival in breast cancer patients, suggesting that LMW isoforms can be used as a prognostic factor at the time of diagnosis [90,91,94]. In this study, we showed that progestins increase cyclin E expression at both mRNA and protein levels and that silencing of PR, or of its downstream targets Stat3 and c-Myc, inhibited this increase. Other authors have already shown that cyclin E is a target of miR-16 [38] in different models. However, our study is the first to show the relevance of miR-16 modulation in breast cancer models throughout a stimulus that is relevant to breast cancer pathophysiology. A role for miR-16 has also been shown in ovarian cancer. Interestingly, patient samples displayed lower levels of miR-16 compared with ovarian surface epithelial cells, the decrease being associated with an augmented cancer cell proliferation and clonal growth. The authors showed that the miR-16 target protein responsible for the proliferative effect in ovarian cancer was the oncogenic protein Bmi-1 [95].

Here, we have also demonstrated the role of miR-16 in progestin-induced breast cancer cell proliferation. *In vitro *proliferation was abolished by transfection with a miR-16 precursor and, more importantly, *in vivo *expression of miR-16 resulted in the development of smaller tumors, with a growth rate significantly lower than those of the tumors from the control group. These inhibitory effects on proliferation may be due, at least in part, to the capacity of miR-16 to inhibit cyclin E. In fact, we observed via immunohistochemistry that pre-miR-16-C4HD tumors expressed significantly lower levels of cyclin E as compared to pre-miR-CTRL-C4HD tumors. However, even though the role of cyclin E in proliferation is certain, we hypothesize that other target proteins may also be involved in these effects. In this sense, comprehensive characterization of the genes modulated by MPA through miRNAs would be necessary to completely elucidate the mechanism responsible for miR-16-mediated tumor suppression. We consider that the wide range of mRNAs and, therefore, proteins, presumably targeted by miR-16 explains the large effects that a relatively modest decrease in its levels has on cell fate.

In this study, we also showed evidence that HRG modulates miR-16 in the context of HRG-induced breast cancer cell proliferation. HRG is a ligand of the ErbB receptor family, and to our knowledge, this is the first report of an HRG-induced miRNA modulation in breast cancer. Previous papers focused on miRNA modulation elicited by the ErbB receptors, but not the ligand. For example, overexpression of the ErbB-2 receptor caused an increase in the oncogenic miR-21 that conferred an aggressive breast cancer phenotype via the downregulation of the metastasis suppressor protein PDCD-4 [32]. Our results demonstrate that HRG induces a similar mechanism to the one induced by progestins upstream of miR-16 downregulation, that is, activation of Stat3 and upregulation of c-Myc. Our results suggest that miR-16 is a common regulator of cell fate in the mechanisms of steroid hormone or growth factor modulation of breast cancer cell proliferation. In this sense, it is worth mentioning a study which came out during the preparation of this manuscript showing that estradiol induces proliferation and upregulation of survival genes in breast cancer cells, through the repression of several miRNA, among them miR-16 [96].

In addition to identifying a new mechanism of action for progestin in breast cancer, our results suggest that miR-16 may be considered a candidate for targeted breast cancer treatment. A miR-16-based treatment would have the potential to target multiple genes and pathways, thereby amplifying the antiproliferative response. Most of the current approaches aimed at targeting miRNAs were developed to interfere with or block miRNA functions. So far, nanoparticles are one of the few formulations that have been used successfully for *in vitro *delivery of small RNA particles (primarily siRNAs). However, the translation from *in vitro *to *in vivo *delivery systems remains a work in progress. Establishing ideal organ-specific delivery systems, while minimizing toxicity and off-target effects will be essential to moving the field forward [97].

## Conclusions

The results of this study demonstrate that progestins modulate a subset of the miRNAs expressed in breast cancer. Importantly, the tumor suppressor miR-16 was among the downregulated miRNAs, and the cell cycle promoter protein cyclin E was identified as one of its targets (Figure 7). Forced expression of miR-16 in tumor cells proved to be an efficient means to slow down tumor growth. Our results shed light on the role of miRNAs in steroid hormone receptor-positive breast cancer and provide potential new targets for future therapeutic approaches.

## Abbreviations

Bp: base pair; CCNE1: cyclin E1; ChIP: chromatin immuoprecipitation; DMEM: Dulbecco's modified Eagle's medium; FCS: fetal calf serum; HRG: heregulin; miRNA: microRNA; MPA: medroxyprogesterone acetate; PR: progesterone receptor; PRE: progesterone response element; RT-qPCR: reverse transcriptase quantitative polymerase chain reaction; siRNA: small interfering RNA; Stat3: signal transducer and activator of transcription 3; UTR: untranslated region.

## Competing interests

The authors declare that they have no competing interests.

## Authors' contributions

MAR designed and performed the experiments, assembled and analyzed the data, and prepared the manuscript. LV and Y-WH performed experiments and participated in data analysis. RS analyzed the data. TH-MH participated in the design of the study and analyzed data. PVE conceived and designed the study, analyzed data, supervised the research, and prepared the manuscript. All authors read and approved the final manuscript.

## Supplementary Material

Additional file 1 **Efficiency of siRNA to PR, Stat3 and c-Myc**. The western blot (WB) in the left panel shows the effect of PR siRNA #1 and #2 on PR expression in C4HD cells. Middle panel shows the effect of Stat3 siRNA #1 and #3 on Stat3 expression on C4HD cells. Right panel shows the effect of c-Myc siRNA #5 and #6 on c-Myc expression on C4HD cells. Click here for file

Additional file 2 **Candidate miR-16 target genes assessed by RT-qPCR**. The official name and the function of the predicted miR-16 target genes that were assessed by RT-qPCR are shown. In the Primers column, F indicates the sequence of the forward primer used and R the one of the reverse. Click here for file

Additional file 3 **MPA modulates miRNAs in murine breast cancer C4HD cells**. The full list of miRNAs expressed in C4HD cells, as assessed by Applied Biosystems Mouse Low Density qPCR miRNA Array A and B cards. In total, 585 miRNAs were surveyed; however, only the 350 miRNAs that were expressed in at least one condition (CTRL or MPA) are shown here. The fold change in expression between the MPA and CTRL conditions is shown to the right of the official name for each miRNA. Click here for file
