# Supplementary material for: Downregulation of the tumor-suppressor miR-16 via progestin-mediated oncogenic signaling contributes to breast cancer development
Source: Breast Cancer Res. 2012 May 14;14(3):R77. doi: 10.1186/bcr3187 (PMC3446340; doi:10.1186/bcr3187)
Supplement: Additional file 2 — Candidate miR-16 target genes assessed by RT-qPCR. The official name and the function of the predicted miR-16 target genes that were assessed by RT-qPCR are shown. In the Primers column, F indicates the sequence of the forward primer used and R the one of the reverse. [file bcr3187-S2.DOCX]

| **Gene symbol** | **Official full name** | **Function** | **Primers** | **Refs** |
| --- | --- | --- | --- | --- |
| RAP2C | Member of RAS oncogene family | RAS domain-containing, small GTP-binding protein | F: actgcaccaaagctctcgat  R: taaacttgccacacccttcc | [1] |
| RAF1 | v-raf-1 murine leukemia viral oncogene homolog 1 | MAP kinase kinase kinase (MAP3K), functions downstream of the Ras family of membrane-associated GTPases | F: caggaagacaaggggattga  R: gtatcccaggctggtcttga | [2] |
| CCNT2 | Cyclin T2 | regulator of CDK kinases, subunit of the transcription elongation factor p-TEFb | F: cctccagtgcaaggaagaag  R: gagggggtaagggatggtta | [3] |
| TCFAP2D | Transcription factor AP-2 delta | member of the AP-2 family of transcription factors, expressed during embryogenesis | F: taagtgggtacgaggcaagg  R: tgatgggtttctccaaaagc | [4] |
| BCL2L2 | BCL2-like 2, Bcl-w | reduces cell apoptosis under cytotoxic conditions | F: caggaaccagggtcaggtta  R: cctggttttccctgacagaa | [5] |
| CCNE1 | Cyclin E1 | regulator of CDK kinases, activity required for cell cycle G1/S transition | F: caccactgagtgctccagaa  R: ctgttggctgacagtggaga | [6] |
| TRAF3 | TNF receptor-associated factor 3 | critical component for NF-kappaB activation | F: aggaggttacaagcccaggt  R: gagcgcccacagattcttag | [7] |
| Akt3 | v-akt murine thymoma viral oncogene homolog 3 | kinase known to regulate cell signaling in response to growth factors, involved in tumorigenesis | F: gaaactggccacttctgctc  R: actgaggtgtggtggagacc | [8] |
| DMTF1 | Cyclin D binding myb-like transcription factor 1 | transcription factor, induced by Ras oncogene | F: cagatgcctaggttccttgc  R: aggcaggtggctcatttcta | [9] |
| WNT3A | Wingless-type MMTV integration site family, member 3A | secreted signaling protein, implicated in oncogenesis and in several developmental processes | F: ccctttccagtcctggtgta  R: cttgaagaaggggtgcagag | [10] |
| RREB1 | Ras responsive element binding protein 1 | zinc finger transcription factor, binds to RAS-responsive elements (RREs) in promoters | F: tgtcagacctgtgagcgaac  R: ggtagcactgtgggtggact | [11] |
| BDNF | Brain-derived neurotrophic factor. | member of the nerve growth factor family | F: cagtggctggctctcttacc  R: tgctgccatgcataaaacat | [12] |

**Additional file 2. Candidate miR-16 target genes assessed by RT-qPCR in Figure 4B.**

References

[1] S. Paganini, G. F. Guidetti, S. Catricala, P. Trionfini, S. Panelli, C. Balduini and M. Torti, *Identification and biochemical characterization of Rap2C, a new member of the Rap family of small GTP-binding proteins*. Biochimie *88,* 285-295 (2006).

[2] A. S. Oh, L. A. Lorant, J. N. Holloway, D. L. Miller, F. G. Kern and D. El Ashry, *Hyperactivation of MAPK induces loss of ERalpha expression in breast cancer cells*. Mol Endocrinol *15,* 1344-1359 (2001).

[3] J. Kohoutek, Q. Li, D. Blazek, Z. Luo, H. Jiang and B. M. Peterlin, *Cyclin T2 is essential for mouse embryogenesis*. Mol Cell Biol *29,* 3280-3285 (2009).

[4] C. Cheng, K. Ying, M. Xu, W. Zhao, Z. Zhou, Y. Huang, W. Wang, J. Xu, L. Zeng, Y. Xie and Y. Mao, *Cloning and characterization of a novel human transcription factor AP-2 beta like gene (TFAP2BL1)*. Int J Biochem Cell Biol *34,* 78-86 (2002).

[5] L. Gibson, S. P. Holmgreen, D. C. Huang, O. Bernard, N. G. Copeland, N. A. Jenkins, G. R. Sutherland, E. Baker, J. M. Adams and S. Cory, *bcl-w, a novel member of the bcl-2 family, promotes cell survival*. Oncogene *13,* 665-675 (1996).

[6] K. Keyomarsi, S. L. Tucker, T. A. Buchholz, M. Callister, Y. Ding, G. N. Hortobagyi, I. Bedrosian, C. Knickerbocker, W. Toyofuku, M. Lowe, T. W. Herliczek and S. S. Bacus, *Cyclin E and survival in patients with breast cancer*. N Engl J Med *347,* 1566-1575 (2002).

[7] I. Aronchik, L. F. Bjeldanes and G. L. Firestone, *Direct inhibition of elastase activity by indole-3-carbinol triggers a CD40-TRAF regulatory cascade that disrupts NF-kappaB transcriptional activity in human breast cancer cells*. Cancer Res *70,* 4961-4971 (2010).

[8] K. Nakatani, D. A. Thompson, A. Barthel, H. Sakaue, W. Liu, R. J. Weigel and R. A. Roth, *Up-regulation of Akt3 in estrogen receptor-deficient breast cancers and androgen-independent prostate cancer lines*. J Biol Chem *274,* 21528-21532 (1999).

[9] R. Sreeramaneni, A. Chaudhry, M. McMahon, C. J. Sherr and K. Inoue, *Ras-Raf-Arf signaling critically depends on the Dmp1 transcription factor*. Mol Cell Biol *25,* 220-232 (2005).

[10] M. Katoh, *Regulation of WNT3 and WNT3A mRNAs in human cancer cell lines NT2, MCF-7, and MKN45*. Int J Oncol *20,* 373-377 (2002).

[11] N. K. Mukhopadhyay, B. Cinar, L. Mukhopadhyay, M. Lutchman, A. S. Ferdinand, J. Kim, L. W. Chung, R. M. Adam, S. K. Ray, A. B. Leiter, J. P. Richie, B. C. Liu and M. R. Freeman, *The zinc finger protein ras-responsive element binding protein-1 is a coregulator of the androgen receptor: implications for the role of the Ras pathway in enhancing androgenic signaling in prostate cancer*. Mol Endocrinol *21,* 2056-2070 (2007).

[12] E. Vanhecke, E. Adriaenssens, S. Verbeke, S. Meignan, E. Germain, N. Berteaux, V. Nurcombe, B. Le, X and H. Hondermarck, *Brain-derived neurotrophic factor and neurotrophin-4/5 are expressed in breast cancer and can be targeted to inhibit tumor cell survival*. Clin Cancer Res *17,* 1741-1752 (2011).
