# Supplementary material for: Downregulation of the tumor-suppressor miR-16 via progestin-mediated oncogenic signaling contributes to breast cancer development
Source: Breast Cancer Res. 2012 May 14;14(3):R77. doi: 10.1186/bcr3187 (PMC3446340; doi:10.1186/bcr3187)
Supplement: Additional file 3 — MPA modulates miRNAs in murine breast cancer C4HD cells. The full list of miRNAs expressed in C4HD cells, as assessed by Applied Biosystems Mouse Low Density qPCR miRNA Array A and B cards. In total, 585 miRNAs were surveyed; however, only the 350 miRNAs that were expressed in at least one condition (CTRL or MPA) are shown here. The fold change in expression between the MPA and CTRL conditions is shown to the right of the official name for each miRNA. [file bcr3187-S3.DOCX]

**Additional file 3. MPA modulates miRNAs in murine breast cancer C4HD cells.**

| **miR** |  | **fold change (MPA vs. CTRL)** |
| --- | --- | --- |
| mmu-miR-410 |  | -14,75 |
| mmu-miR-379 |  | -12,91 |
| mmu-miR-582-5p |  | -10,51 |
| rno-miR-450a |  | -9,21 |
| mmu-miR-101a* |  | -7,85 |
| rno-miR-381 |  | -6,99 |
| mmu-miR-215 |  | -6,38 |
| mmu-miR-378* |  | -6,28 |
| mmu-miR-376a |  | -5,39 |
| mmu-miR-224 |  | -5,01 |
| mmu-miR-682 |  | -4,74 |
| rno-miR-190b |  | -4,08 |
| mmu-miR-376b* |  | -4,05 |
| mmu-miR-24-1* |  | -4,04 |
| mmu-miR-196a* |  | -3,71 |
| mmu-miR-539 |  | -3,34 |
| mmu-miR-542-3p |  | -3,10 |
| rno-miR-136* |  | -2,95 |
| mmu-miR-294 |  | -2,89 |
| mmu-miR-411 |  | -2,53 |
| mmu-miR-295 |  | -2,44 |
| rno-miR-532-5p |  | -2,31 |
| mmu-miR-875-5p |  | -2,25 |
| mmu-miR-376c |  | -2,12 |
| mmu-miR-34b-3p |  | -2,08 |
| mmu-miR-16 |  | -1,98 |
| mmu-miR-200c* |  | -1,91 |
| mmu-miR-674 |  | -1,82 |
| mmu-miR-362-3p |  | -1,79 |
| mmu-miR-547 |  | -1,75 |
| mmu-miR-197 |  | -1,67 |
| mmu-miR-497 |  | -1,67 |
| mmu-let-7f* |  | -1,66 |
| mmu-miR-715 |  | -1,62 |
| mmu-miR-202-3p |  | -1,61 |
| rno-miR-28* |  | -1,61 |
| mmu-miR-15a |  | -1,60 |
| mmu-miR-195 |  | -1,56 |
| mmu-miR-29c* |  | -1,56 |
| mmu-miR-92a* |  | -1,53 |
| mmu-miR-30c-1* |  | -1,51 |
| mmu-miR-181a-1* |  | -1,50 |
| mmu-miR-7b |  | -1,49 |
| rno-miR-339-3p |  | -1,49 |
| mmu-miR-193 |  | -1,49 |
| mmu-let-7i* |  | -1,45 |
| mmu-miR-135b |  | -1,40 |
| mmu-miR-152 |  | -1,38 |
| mmu-miR-214 |  | -1,38 |
| mmu-miR-721 |  | -1,38 |
| mmu-miR-148b |  | -1,37 |
| mmu-miR-324-3p |  | -1,36 |
| mmu-miR-687 |  | -1,36 |
| mmu-miR-138 |  | -1,35 |
| mmu-miR-351 |  | -1,35 |
| mmu-miR-181c |  | -1,34 |
| mmu-miR-361 |  | -1,33 |
| mmu-miR-503 |  | -1,31 |
| mmu-miR-592 |  | -1,31 |
| mmu-miR-125b-5p |  | -1,31 |
| mmu-miR-136 |  | -1,31 |
| mmu-miR-744 |  | -1,30 |
| mmu-miR-574-3p |  | -1,30 |
| mmu-miR-339-3p |  | -1,29 |
| mmu-miR-125a-5p |  | -1,28 |
| mmu-miR-100 |  | -1,28 |
| mmu-miR-764-5p |  | -1,28 |
| mmu-miR-106b* |  | -1,24 |
| mmu-miR-345-5p |  | -1,24 |
| mmu-miR-125a-3p |  | -1,23 |
| mmu-miR-301a |  | -1,23 |
| mmu-miR-138* |  | -1,23 |
| mmu-miR-187 |  | -1,22 |
| mmu-miR-615-3p |  | -1,21 |
| mmu-miR-200c |  | -1,20 |
| mmu-miR-128a |  | -1,19 |
| rno-miR-743a |  | -1,19 |
| mmu-miR-155 |  | -1,19 |
| mmu-miR-31 |  | -1,19 |
| mmu-miR-328 |  | -1,19 |
| mmu-miR-467d |  | -1,18 |
| mmu-miR-324-5p |  | -1,17 |
| mmu-miR-339-5p |  | -1,17 |
| mmu-miR-805 |  | -1,17 |
| mmu-miR-467e |  | -1,16 |
| mmu-miR-10a* |  | -1,16 |
| mmu-miR-206 |  | -1,16 |
| mmu-miR-340-5p |  | -1,16 |
| mmu-miR-322 |  | -1,15 |
| mmu-miR-500 |  | -1,15 |
| mmu-miR-199a-3p |  | -1,15 |
| mmu-miR-186* |  | -1,15 |
| mmu-miR-26b* |  | -1,15 |
| mmu-miR-484 |  | -1,15 |
| mmu-miR-337-3p |  | -1,15 |
| mmu-miR-674* |  | -1,14 |
| mmu-miR-26a |  | -1,14 |
| mmu-miR-200b |  | -1,14 |
| mmu-miR-532-3p |  | -1,14 |
| mmu-miR-146b* |  | -1,13 |
| mmu-miR-760 |  | -1,13 |
| mmu-miR-19a |  | -1,13 |
| mmu-miR-149 |  | -1,13 |
| mmu-miR-30c-2* |  | -1,12 |
| mmu-miR-872* |  | -1,12 |
| mmu-miR-143 |  | -1,12 |
| mmu-miR-222 |  | -1,12 |
| mmu-miR-210 |  | -1,12 |
| mmu-miR-16* |  | -1,11 |
| mmu-miR-99b |  | -1,11 |
| mmu-miR-327 |  | -1,11 |
| mmu-miR-467b |  | -1,11 |
| mmu-miR-30c |  | -1,11 |
| mmu-miR-671-3p |  | -1,11 |
| mmu-miR-30d |  | -1,10 |
| mmu-miR-34a |  | -1,10 |
| mmu-miR-221 |  | -1,10 |
| mmu-miR-151-3p |  | -1,10 |
| rno-miR-30d* |  | -1,10 |
| mmu-miR-15b |  | -1,09 |
| mmu-miR-331-3p |  | -1,09 |
| mmu-miR-297b-5p |  | -1,09 |
| mmu-miR-23b |  | -1,09 |
| mmu-miR-872 |  | -1,09 |
| mmu-miR-200a* |  | -1,09 |
| mmu-miR-30e |  | -1,08 |
| mmu-let-7e |  | -1,08 |
| mmu-miR-21 |  | -1,08 |
| mmu-miR-191 |  | -1,08 |
| mmu-miR-184 |  | -1,08 |
| mmu-let-7a |  | -1,08 |
| rno-miR-352 |  | -1,08 |
| mmu-miR-186 |  | -1,08 |
| mmu-miR-33* |  | -1,07 |
| mmu-miR-146a |  | -1,07 |
| mmu-miR-30a |  | -1,07 |
| mmu-miR-10a |  | -1,07 |
| mmu-miR-342-3p |  | -1,07 |
| mmu-miR-343 |  | -1,07 |
| mmu-miR-877* |  | -1,07 |
| mmu-miR-15b* |  | -1,06 |
| mmu-miR-27b* |  | -1,06 |
| mmu-miR-466b-3-3p |  | -1,06 |
| mmu-miR-148a |  | -1,06 |
| mmu-miR-31* |  | -1,06 |
| mmu-miR-130b |  | -1,06 |
| mmu-miR-22* |  | -1,06 |
| mmu-miR-685 |  | -1,06 |
| mmu-miR-146b |  | -1,05 |
| mmu-miR-28 |  | -1,05 |
| mmu-miR-193* |  | -1,05 |
| mmu-let-7d |  | -1,05 |
| mmu-miR-302a* |  | -1,05 |
| mmu-let-7c |  | -1,05 |
| mmu-miR-196b |  | -1,05 |
| mmu-miR-99a |  | -1,05 |
| mmu-miR-140 |  | -1,05 |
| mmu-miR-200a |  | -1,04 |
| mmu-miR-20b |  | -1,04 |
| mmu-miR-30b |  | -1,04 |
| mmu-let-7g |  | -1,04 |
| mmu-miR-26b |  | -1,04 |
| mmu-let-7b |  | -1,04 |
| mmu-miR-30b* |  | -1,04 |
| mmu-miR-878-3p |  | -1,03 |
| mmu-miR-214* |  | -1,03 |
| mmu-miR-467c |  | -1,03 |
| mmu-miR-503* |  | -1,03 |
| mmu-miR-193b |  | -1,03 |
| mmu-miR-10b* |  | -1,03 |
| mmu-miR-7a* |  | -1,03 |
| mmu-miR-96 |  | -1,03 |
| mmu-miR-546 |  | -1,03 |
| mmu-miR-29a* |  | -1,03 |
| mmu-let-7f |  | -1,03 |
| mmu-miR-34c* |  | -1,03 |
| mmu-miR-425 |  | -1,03 |
| mmu-miR-145 |  | -1,02 |
| mmu-miR-20a |  | -1,02 |
| rno-miR-196c |  | -1,02 |
| mmu-miR-27b |  | -1,02 |
| mmu-miR-188-5p |  | -1,02 |
| mmu-miR-29b* |  | -1,02 |
| mmu-miR-103 |  | -1,01 |
| mmu-miR-700 |  | -1,01 |
| mmu-let-7a* |  | -1,01 |
| mmu-miR-19b |  | -1,01 |
| mmu-miR-301b |  | -1,01 |
| mmu-miR-24 |  | -1,01 |
| mmu-miR-98 |  | -1,01 |
| mmu-miR-218-1* |  | -1,01 |
| mmu-miR-203 |  | -1,00 |
| mmu-miR-10b |  | -1,00 |
| mmu-miR-183* |  | -1,00 |
| mmu-miR-22 |  | -1,00 |
| mmu-miR-652 |  | -1,00 |
| mmu-miR-141 |  | 1,00 |
| mmu-miR-7a |  | 1,01 |
| rno-miR-489 |  | 1,01 |
| mmu-miR-429 |  | 1,01 |
| mmu-let-7i |  | 1,01 |
| mmu-miR-491 |  | 1,01 |
| mmu-miR-24-2* |  | 1,01 |
| mmu-miR-9 |  | 1,01 |
| mmu-miR-297a* |  | 1,01 |
| mmu-miR-30e* |  | 1,02 |
| mmu-miR-330 |  | 1,02 |
| mmu-miR-93* |  | 1,02 |
| mmu-miR-720 |  | 1,02 |
| mmu-miR-29a |  | 1,02 |
| mmu-miR-132 |  | 1,02 |
| mmu-miR-27a |  | 1,02 |
| mmu-miR-126-3p |  | 1,02 |
| rno-miR-351 |  | 1,02 |
| mmu-miR-467a* |  | 1,03 |
| mmu-miR-466a-3p |  | 1,03 |
| mmu-miR-699 |  | 1,03 |
| mmu-miR-93 |  | 1,03 |
| mmu-miR-320 |  | 1,04 |
| mmu-miR-200b* |  | 1,04 |
| mmu-miR-30a* |  | 1,05 |
| mmu-miR-130b* |  | 1,05 |
| mmu-miR-29b |  | 1,05 |
| mmu-miR-29c |  | 1,05 |
| mmu-miR-374 |  | 1,05 |
| mmu-miR-340-3p |  | 1,05 |
| mmu-miR-106b |  | 1,05 |
| mmu-miR-690 |  | 1,05 |
| mmu-miR-182 |  | 1,05 |
| mmu-miR-378 |  | 1,05 |
| mmu-miR-192 |  | 1,05 |
| mmu-miR-801 |  | 1,06 |
| mmu-miR-183 |  | 1,06 |
| mmu-miR-542-5p |  | 1,06 |
| mmu-miR-122 |  | 1,06 |
| mmu-miR-181a |  | 1,06 |
| mmu-miR-709 |  | 1,07 |
| mmu-miR-101a |  | 1,07 |
| mmu-miR-32 |  | 1,07 |
| mmu-miR-15a* |  | 1,07 |
| rno-miR-463 |  | 1,07 |
| mmu-miR-190 |  | 1,07 |
| mmu-miR-106a |  | 1,07 |
| mmu-miR-23a |  | 1,08 |
| rno-miR-7a* |  | 1,08 |
| mmu-miR-758 |  | 1,09 |
| mmu-miR-678 |  | 1,09 |
| mmu-miR-130a |  | 1,10 |
| mmu-miR-365 |  | 1,10 |
| mmu-miR-25 |  | 1,10 |
| mmu-miR-466d-3p |  | 1,10 |
| mmu-miR-101b |  | 1,10 |
| mmu-miR-141* |  | 1,10 |
| mmu-miR-20a* |  | 1,11 |
| mmu-miR-139-5p |  | 1,11 |
| mmu-miR-133a |  | 1,11 |
| mmu-miR-92a |  | 1,12 |
| mmu-miR-17 |  | 1,12 |
| mmu-miR-532-5p |  | 1,12 |
| mmu-miR-28* |  | 1,12 |
| rno-miR-207 |  | 1,13 |
| mmu-let-7d* |  | 1,13 |
| mmu-miR-494 |  | 1,14 |
| mmu-miR-338-3p |  | 1,14 |
| mmu-miR-27a* |  | 1,15 |
| mmu-miR-135a* |  | 1,15 |
| mmu-miR-704 |  | 1,15 |
| mmu-miR-669a |  | 1,15 |
| mmu-miR-199b* |  | 1,17 |
| mmu-miR-21* |  | 1,17 |
| mmu-miR-701 |  | 1,17 |
| mmu-miR-467a |  | 1,18 |
| mmu-miR-18a* |  | 1,18 |
| mmu-miR-194 |  | 1,18 |
| mmu-miR-19a* |  | 1,19 |
| mmu-miR-142-3p |  | 1,19 |
| mmu-miR-467b* |  | 1,21 |
| mmu-miR-672 |  | 1,22 |
| mmu-miR-18a |  | 1,23 |
| mmu-miR-450a-5p |  | 1,24 |
| rno-miR-664 |  | 1,24 |
| mmu-miR-375 |  | 1,24 |
| mmu-miR-409-3p |  | 1,24 |
| mmu-miR-190b |  | 1,25 |
| rno-miR-99a* |  | 1,25 |
| mmu-miR-150* |  | 1,26 |
| mmu-miR-330* |  | 1,26 |
| mmu-miR-692 |  | 1,31 |
| mmu-miR-350 |  | 1,33 |
| mmu-miR-211 |  | 1,34 |
| mmu-miR-804 |  | 1,34 |
| mmu-miR-744* |  | 1,34 |
| mmu-miR-145* |  | 1,35 |
| mmu-miR-185 |  | 1,35 |
| mmu-miR-673-5p |  | 1,35 |
| mmu-miR-667 |  | 1,35 |
| mmu-miR-299* |  | 1,37 |
| mmu-miR-127 |  | 1,38 |
| mmu-miR-694 |  | 1,38 |
| rno-miR-20b-5p |  | 1,38 |
| mmu-miR-322* |  | 1,39 |
| mmu-miR-877 |  | 1,40 |
| mmu-miR-9* |  | 1,42 |
| mmu-miR-676* |  | 1,43 |
| mmu-miR-706 |  | 1,44 |
| mmu-miR-763 |  | 1,45 |
| mmu-miR-466d-5p |  | 1,51 |
| mmu-miR-223 |  | 1,52 |
| mmu-miR-147 |  | 1,53 |
| mmu-miR-326 |  | 1,53 |
| mmu-miR-99b* |  | 1,57 |
| rno-miR-1 |  | 1,57 |
| rno-let-7e* |  | 1,61 |
| rno-miR-345-3p |  | 1,65 |
| mmu-miR-501-5p |  | 1,65 |
| mmu-miR-129-3p |  | 1,75 |
| mmu-miR-467e* |  | 1,86 |
| mmu-let-7g* |  | 1,96 |
| mmu-miR-296-5p |  | 2,07 |
| mmu-miR-1 |  | 2,07 |
| mmu-miR-680 |  | 2,09 |
| mmu-miR-17* |  | 2,13 |
| mmu-miR-425* |  | 2,16 |
| mmu-miR-126-5p |  | 2,22 |
| rno-miR-148b-5p |  | 2,25 |
| mmu-miR-331-5p |  | 2,34 |
| mmu-miR-455* |  | 2,58 |
| mmu-miR-434-5p |  | 2,85 |
| mmu-miR-423-5p |  | 3,03 |
| mmu-miR-191* |  | 3,47 |
| mmu-miR-376b |  | 3,54 |
| mmu-miR-466h |  | 3,54 |
| rno-miR-224 |  | 4,58 |
| rno-miR-493 |  | 6,22 |
| mmu-miR-698 |  | 7,34 |
| mmu-miR-470* |  | 8,15 |
| mmu-miR-708 |  | 8,61 |
| rno-miR-29b-2* |  | 9,28 |
| mmu-miR-297c |  | 10,98 |
| mmu-miR-451 |  | 11,81 |
| mmu-miR-702 |  | 12,47 |
| mmu-miR-218 |  | 14,54 |
| rno-miR-23a* |  | 29,01 |
| mmu-miR-434-3p |  | 39,33 |
| mmu-miR-485* |  | 193,43 |
